# Prolonged lifespan of initial-anode-free lithium-metal battery by pre-lithiation in Li-rich Li_2_Ni_0.5_Mn_1.5_O_4_ spinel cathode†

**DOI:** 10.1039/d2sc06772b

**Published:** 2023-01-25

**Authors:** Leiyu Chen, Chao-Lung Chiang, Xiaohong Wu, Yonglin Tang, Guifan Zeng, Shiyuan Zhou, Baodan Zhang, Haitang Zhang, Yawen Yan, Tingting Liu, Hong-Gang Liao, Xiaoxiao Kuai, Yan-Gu Lin, Yu Qiao, Shi-Gang Sun

**Affiliations:** a State Key Laboratory of Physical Chemistry of Solid Surfaces, iChEM (Collaborative Innovation Center of Chemistry for Energy Materials), Department of Chemistry, College of Chemistry and Chemical Engineering, Xiamen University Xiamen 361005 PR China kuaixiaoxiao@xmu.edu.cn yuqiao@xmu.edu.cn; b National Synchrotron Radiation Research Center Hsinchu 30076 Taiwan Republic of China lin.yg@nsrrc.org.tw; c Fujian Science & Technology Innovation Laboratory for Energy Materials of China (Tan Kah Kee Innovation Laboratory) Xiamen 361005 PR China; d School of Environmental Science and Engineering, Suzhou University of Science and Technology Suzhou 215009 China

## Abstract

Anode-free lithium metal batteries (AF-LMBs) can deliver the maximum energy density. However, achieving AF-LMBs with a long lifespan remains challenging because of the poor reversibility of Li^+^ plating/stripping on the anode. Here, coupled with a fluorine-containing electrolyte, we introduce a cathode pre-lithiation strategy to extend the lifespan of AF-LMBs. The AF-LMB is constructed with Li-rich Li_2_Ni_0.5_Mn_1.5_O_4_ cathodes as a Li-ion extender; the Li_2_Ni_0.5_Mn_1.5_O_4_ can deliver a large amount of Li^+^ in the initial charging process to offset the continuous Li^+^ consumption, which benefits the cycling performance without sacrificing energy density. Moreover, the cathode pre-lithiation design has been practically and precisely regulated using engineering methods (Li-metal contact and pre-lithiation Li-biphenyl immersion). Benefiting from the highly reversible Li metal on the Cu anode and Li_2_Ni_0.5_Mn_1.5_O_4_ cathode, the further fabricated anode-free pouch cells achieve 350 W h kg^−1^ energy density and 97% capacity retention after 50 cycles.

## Abstract 2

Benefiting from highly reversible structure evolution of pre-lithiated Li-rich Li_2_Ni_0.5_Mn_1.5_O_4_ cathode, the corresponding anode-free pouch cell delivers a considerable energy density of 350 W h kg^−1^ and 97% capacity retention after 50 cycles.

## Graphical abstract

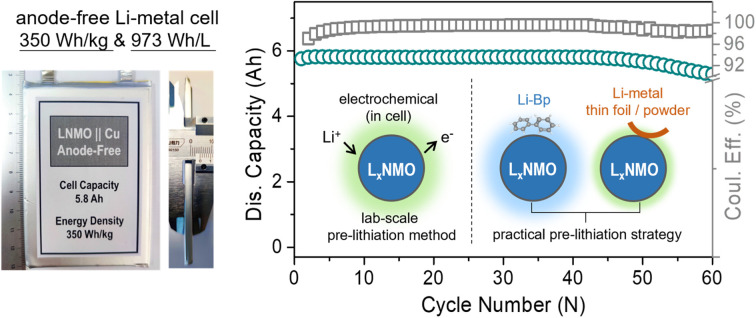

## Introduction

Anode-free lithium metal batteries (AF-LMBs) can maximize the energy density of rechargeable batteries.^1–6^ However, the AF-LMBs suffer from rapid lithium stock loss and poor cycle life, due to the poor reversibility of lithium plating/stripping on the anode.^7–9^ Many approaches have been applied to overcome these limitations, and some promising results have been achieved. Outstanding performance has been achieved using some functional electrolytes; the electrolyte salts were observed to be continuously consumed during cycling, and the limited reversibility of lithium plating/stripping was prominently enhanced.^10–14^ In addition, pre-lithiation could directly lessen the consumption of active Li^+^ during the solid electrolyte interphase (SEI) formation and the continuous consumption of Li^+^ in subsequent cycling.^15,16^ Additionally, Li^+^ cannot intercalate into Cu, so pre-lithiation strategies involving the anode are not suitable for AF-LMBs, which have distinctly different pre-lithiation strategies from those of graphite, Si, and carbon-based anodes.^17–20^

In AF-LMBs, the addition of external lithium sources to the cathode electrode is usually conducted to offset the irreversible capacity loss.^21,22^ This strategy can be divided into two methods: (1) the addition of sacrificial salts (such as Li_3_N, Li_2_O, Li_2_S, Li_2_C_2_O_4_, *etc.*) to the cathode.^23–28^ This method allows precise control of the amount of additional lithium source used. However, these sacrificial salts are not only easily oxidized to generate gases (*e.g.*, N_2_, O_2_, CO_2_, *etc.*), but also remain in the cell, affecting the energy density.^29–31^ (2) The cathode pre-lithiation method. More recently, over-lithiated cathodes that can store extra Li^+^ in the cathode structure have emerged as potential AF-LMBs cathodes because they offset the irreversible capacity loss. The over-lithiated cathode (also called a “Li reservoir”) has crystallographic vacancies that can be occupied by extra Li^+^ and deliver much more extra Li^+^ in the first charging process.^32–34^ For instance, Li_2_[Ni_0.8_Co_0.1_Mn_0.1_]O_2_ was reported, which stores nearly twice as many lithium ions in the lithium layer to improve energy density.^35^ LiNi_0.5_Mn_1.5_O_4_ (LNMO) is a fascinating high-voltage and structurally stable AF-LMB cathode; however, it suffers from limited cycling performance due to the continuous consumption of active Li^+^.^36,37^ The per-lithiation strategy can significantly extend the service life of AF-LMBs; however, their average coulombic efficiencies (ACEs) cannot reach exciting values. The use of various electrolyte additives to improve the ACE of AF-LMBs has been attempted. For example, the addition of suitable additives to carbonate electrolytes was reported to remarkably enhance the electrochemical performance of a Cu‖NMC111 anode-free cell.^3^ In this context, developing superior functional electrolytes and pre-lithiated cathodes with enhanced structure stability is critical to the application of long-lifespan and high-energy-density AF-LMBs.^1,38^

In this study, a strategy of combining functional electrolytes and pre-lithiation is applied in high-voltage spinel-related Li_1.76_Ni_0.5_Mn_1.5_O_4_ (L_1.76_NMO), which is prepared by over-lithiation of *Fd*3̄*m* type LNMO (Fig. S1 and Table S1†), and found to remarkably extend the cycling performance of Cu‖L_1.76_NMO AF-LMB. Furthermore, the L_2_NMO can deliver a large amount of Li^+^ in the first charging process to offset the continuous lithium ion consumption, which benefits the cycling performance without sacrificing energy density. Moreover, after verifying the structural stability and feasibility of the electrochemical pre-lithiation method by comprehensive characterization (XAS and *in situ* XRD), more practical pre-lithiation strategies have been demonstrated and realized in terms of engineering, *e.g.*, Li-metal contact pre-lithiation and Li-biphenyl immersion pre-lithiation methods. These practical pre-lithiation strategies can precisely regulate and supplement the amount of Li^+^ pre-stored within LNMO by regulating the engineering parameters. Furthermore, the introduction of a fluorine-containing electrolyte additive can further improve the cycling performance of the Cu‖L_1.76_NMO anode-free coin cell; remarkably, Cu‖L_1.76_NMO exhibits a capacity retention (CR) of 95.6% after 50 cycles, which is much better than that of Cu‖LNMO and most state-of-art AF-LMBs. Furthermore, a constructed Cu‖L_1.76_NMO anode-free pouch cell comprising a high-mass-loading (27.53 mg cm^−2^) cathode and bare Cu exhibits a higher CR than the coin cell (97.55%), as well as an exceptional energy density of 350 W h kg^−1^ (973 W h L^−1^).

## Results and discussion

**Fig. 1:**
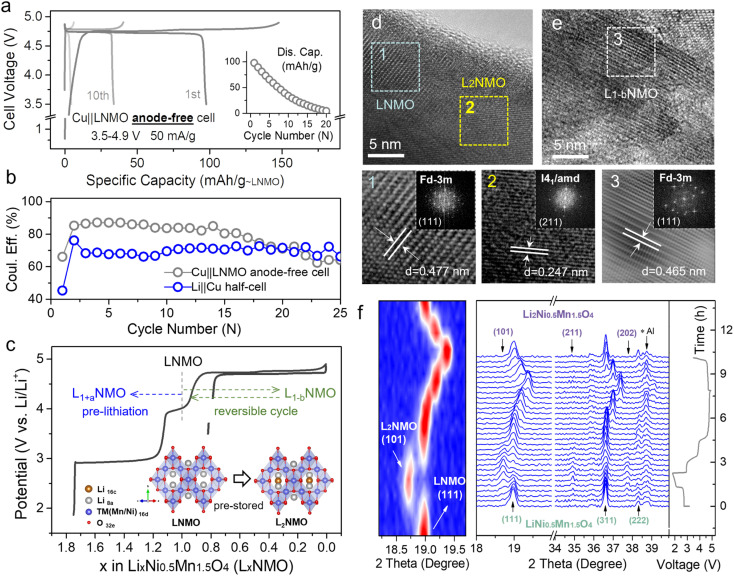
Utilizing the reversible phase transition between LNMO and L_2_NMO for an anode-free cell. (a) Voltage profiles of the Cu‖LNMO anode-free cell for different cycles, and the corresponding cycling performance. (b) Coulombic efficiency *versus* cycle number for the Cu‖LNMO anode-free cell and Li‖Cu half-cell (Fig. S2†). (c) Voltage profiles of the Li‖LNMO half-cell (L_*x*_NMO (0 ≤ *x* < 2)). The inset is a schematic diagram of the reversible phase transition between LNMO and L_2_NMO. HRTEM images of the LNMO electrode (d) discharged to 1.8 V and (e) re-charged to 4.9 V, and the corresponding (inverse) fast Fourier transformation (FFT) patterns of LNMO and L_2_NMO. (f) *In situ* XRD patterns and galvanostatic charge–discharge curve of the Li‖LNMO half-cell (right) and the corresponding contour plot of the (111) main peak of the *in situ* XRD patterns (left). The Li‖LNMO half-cell was discharged from the OCV to 1.8 V, re-charged to 4.9 V, and then re-discharged to 3.5 V at 50 mA g^−1^.

Fig. 1a and S2† show the voltage profiles of the Cu‖LNMO anode-free cell and Li‖Cu half-cell at different cycles. As displayed in Fig. 1b, the Cu‖LNMO anode-free cell has a low initial coulombic efficiency (ICE). Although the coulombic efficiency (CE) of the Cu‖LNMO anode-free cell is improved in the second cycle, its subsequent average coulombic efficiency (ACE) is unsatisfactory. As expected, the capacity of the Cu‖LNMO anode-free cell decreases in the subsequent cycles, and a similar trend is found for the Li‖Cu half-cell. To reveal the capacity decrease mechanism of the Cu‖LNMO anode-free cell in detail, we simulated the influence of the ICE and the ACE on the capacity (Fig. S3†). When the ACE is 99% and different ICE values are considered, the capacity will decay in the subsequent cycles. When the ICE is 100%, the CR after 25 cycles is 78.6%; this CR is substantially greater than those obtained using other ICE values. On the basis of the aforementioned considerations, the capacity decrease of the anode-free cell can be attributed to the following factors. (1) Low ICE. Due to the unavoidable formation of a solid–electrolyte interface (SEI) film on the anode in the first cycle, a large amount of Li will be consumed and a large irreversible capacity will be achieved in the first cycle.^21,39^ Additionally, Li ion plating on the Cu anode will form a large amount of isolated “dead Li”, which can also lead to low ICE.^9^ (2) Low ACE. The continuous consumption of Li^+^ in subsequent cycles and the poor reversibility of lithium plating/stripping lead to a low ACE. The CE *versus* cycle number data for the Li‖LNMO half-cell (Fig. S4†) indicate that the ICE and ACE of the LNMO cathode are superior, which means that the determining factor in the poor cycle stability of the anode-free cell is the Cu anode rather than the LNMO cathode. The practical amount of the additional lithium sources depends on the anode ICE and the subsequent ACE during the cycling process. This general guideline applies to not only anode-free cells, but also graphite-, Si-, and carbon-based full-cells. Many strategies have been applied to overcome the abovementioned issues. One proven strategy to optimize anode-free cells is modification of the liquid electrolyte, which can stabilize the lithium plating/stripping on the anode. However, even if the ACE is improved by optimizing the electrolyte, the ICE will still continuously affect the cycle stability of the anode-free cell, and the capacity will decay in the subsequent cycles under the low-ICE condition. Hence, we combined the modified electrolyte and pre-lithiation strategies to lessen the consumption of active Li^+^ during SEI film formation and offset the irreversible capacity loss and continuous consumption of Li^+^ during subsequent cycling.

LNMO is a fascinating high-voltage and structurally stable AF-LMB cathode; however, it suffers from limited cycling performance due to the continuous consumption of active Li^+^.^36,37^ Surprisingly, the unoccupied octahedral sites of LNMO can store extra Li^+^, thus forming spinel-related Li_2_Ni_0.5_Mn_1.5_O_4_ (L_2_NMO).^5,6,40,41^Fig. 1c shows the charge–discharge curve of the Li‖LNMO half-cell and a schematic diagram of the reversible phase transition between LNMO and L_2_NMO. To further study the phase transition process between LNMO and L_2_NMO, high-resolution transmission electron microscopy (HRTEM) measurements and *in situ* X-ray diffraction (XRD) of the LNMO electrode were conducted during the charge–discharge processes (Fig. 1d–f). The contour plot of the (111) main peak (Fig. 1f left) and corresponding XRD patterns with the corresponding charge–discharge curve in Fig. 1f (right) clearly show the phase transition between LNMO and L_2_NMO. The main peak (111) of *Fd*3̄*m*-type LNMO is gradually split into two peaks, and the characteristic (101), (211), and (202) reflections associated with *I*4_1_/*amd*-type L_2_NMO gradually appear in the first discharge progress, indicating that Li ions are continuously intercalating into the empty octahedral sites and that LNMO partially transforms into L_2_NMO, resulting in the coexistence of LNMO and L_2_NMO.^42^ During the charging process, Li^+^ is extracted from LNMO and L_2_NMO, indicating further delithiation and the generation of L_*x*_NMO (0 ≤ *x* < 2). Upon re-discharge to 3.5 V, all the characteristic reflections of LNMO return to their original positions, suggesting a reversible phase transition between LNMO and L_2_NMO. In addition, when the first discharge cut-off potential is set below 1.8 V, during subsequent charging to 4.9 V, twice as much Li^+^ can be released and temporarily stored on the anode side as a Li reservoir without introducing inactive components at the expense of energy density (Fig. 1c).^23,25,43^ Furthermore, during the re-discharge process, by controlling the discharge cut-off potential, part of the Li^+^ can be stored on the Cu anode, and the other part participates in subsequent battery cycles, therefore extending the anode-free cell life without sacrificing energy density. This was confirmed by the HRTEM images of LNMO at different voltage states (first discharge to 1.8 V and re-charge to 4.9 V) in Fig. 1d and e. The lattice fringes with interlayer spacings of 0.477 and 0.247 nm observed after over-discharge to 1.8 V correspond to the (111) and (211) planes of LNMO and L_2_NMO, indicating the coexistence of LNMO and L_2_NMO. Additionally, after re-charging to 4.9 V the cycled LNMO can still maintain the spinel-like structure, which is consistent with the OCV (Fig. S5†) and over-discharge results, confirming the reversible phase transition between LNMO and L_2_NMO. This result is in line with the *in situ* XRD analyses.

**Fig. 2:**
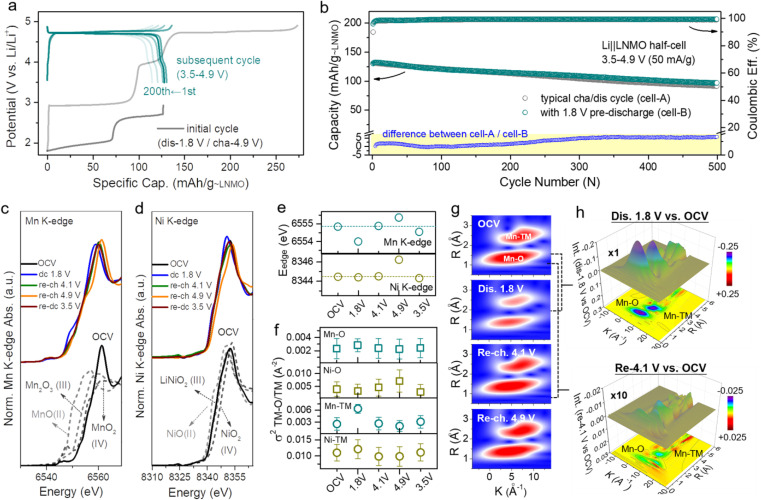
Local structure and TM–O covalent environment analysis of LNMO and L_2_NMO. (a) Voltage profiles of the Li‖L_1.76_NMO half-cell that was first discharged to 1.8 V and then cycled in the voltage range from 3.5 V to 4.9 V at a current density of 50 mA g^−1^ (denoted as cell-B). (b) Cycling performance of cell-A (Fig. S9†) and cell-B, along with the difference between cell-A and cell-B (bottom). (c) Mn K-edge XANES spectra, (d) Ni K-edge XANES spectra, and (e) edge positions of the Mn K-edge and Ni K-edge at different voltage states. (f) Debye–Waller factor *σ*^2^ of the Mn/Ni–O bond and Mn/Ni–TM bond of LNMO. (g) WT-EXAFS spectra of the Mn–O bond and Mn–TM bond at different voltage states. (h) Colour 3D WT-EXAFS spectra showing the difference between the WT-EXAFS spectra of the Mn–O bond and Mn–TM bond (first discharge to 1.8 V *vs.* OCV (top) and re-charge to 4.1 V *vs.* OCV (bottom)).

However, the intercalation of too much extra Li^+^ into the crystal will reduce its structural stability, so it is favorable to use a partly lithiated cathode in practical applications. Fig. 2a (dark line) shows the over-lithiation charge–discharge curve of L_1.76_NMO (Fig. S6–S8 and Table S2†); the specific discharge capacity is 112 mA h g^−1^ to 1.8 V at 50 mA g^−1^ (76% of the theoretical capacity). To investigate the effect of over-lithiation on the electrochemical stability of LNMO, the charge–discharge curves of the Li‖LNMO half-cell (denoted as cell-A, cycled in the range from 3.5 V to 4.9 V) and over-lithiated Li‖L_1.76_NMO half-cell (denoted as cell-B) are shown in Fig. S9† and 2a, respectively. As expected, cell-B delivers a high discharge capacity with 97% CE in the first cycle, which is much better than that of cell-A. The improved ICE of cell-B is expected to be due to the pre-lithiation of LNMO in the first discharge process (Fig. 2b). Additionally, both cell-A and cell-B show excellent cycling stability even after 500 cycles at a current density of 50 mA g^−1^. However, the Li‖L_1.76_NMO half-cell cannot be cycled under a voltage window of 1.8–4.9 V (Fig. S10†), due to the typical Jahn–Teller distortion.^41,44^

The local structure information of atoms is highly related to the electrochemical performance of materials.^45^ X-ray absorption spectroscopy (XAS) is a powerful technique to characterize the local structure around resonant (absorbing) atoms. Each XAS spectrum comprises an X-ray absorption near edge structure (XANES) and extended X-ray absorption fine structure (EXAFS), which are defined by the threshold of the XAS spectrum at the edge-jump region at ±20 eV of the element's K-edge photon energy. To explore the intrinsic structural changes of LNMO after pre-lithiation, we performed *ex situ* XAS at the Mn and Ni K-edges. Fig. 2c–e presents the oxidation state variation of the manganese and nickel species in LNMO during a complete discharge–charge process at several specific voltages (open circuit voltage (OCV), dc 1.8, re-ch 4.1, re-ch 4.9, and re-dc 3.5 V; dc: discharge, re-ch: re-charge). The Mn and Ni K-edge XAS spectra at the OCV were compared with the relevant standards and are shown in the lower portion. The major oxidation states of manganese and nickel in LNMO at the OCV state were identified as Mn(iv) and Ni(ii) with mixed-valence states, respectively. Once the LNMO was discharged to 1.8 V, the edge position of Mn shifted from 6554.8 eV to a lower photon energy of 6554 eV owing to the lithiation-induced valence reduction of manganese from Mn^4+^ to lower valence, leading to the coexistence of LNMO and L_2_NMO (Fig. 2c and e).^46,47^ The edge position shifted back to its initial value when LNMO was at the re-ch 4.1 V state. At the subsequent high re-ch 4.9 V state, the edge position further shifted to 6555.3 eV due to the possible over-delithiation of LNMO. Eventually, the edge position returned to a lower value (6554.5 eV) than the initial one at re-dc 3.5 V, due to slight oxygen vacancy formation. In terms of the nickel species, the variations in the edge position were relatively insignificant (Fig. 2d and e). The edge position remained unchanged at OCV, dc 1.8 V, and re-ch 4.1 V, revealing the obvious stability of the nickel species of LNMO. At the high re-ch 4.9 V state, the edge position shifted from 8344.5 to a higher photon energy of 8346.2 eV, and then finally returned to the original value at re-dc 3.5 V. This edge position variation also depends on the lithiation and structural reversibility. The corresponding points are also presented for reference in Fig. S11.† Furthermore, the Mn and Ni K-edge EXAFS spectra (Fig. S12†) provide the structural variation information of LNMO over the period of a lithiation–delithiation cycle.

A quantitative parameter, the Debye–Waller factor (*σ*^2^), was adopted to estimate the relative structural disorder between the adjacent atoms around the target atom (*i.e.*, Mn and Ni),^48^ as exhibited in Fig. 2f. The interatomic coordination between Mn, Ni, and the adjacent atoms is classified as Mn/Ni–O and Mn/Ni–TM (TM: transition metal, Mn or Ni). In comparison to the original *σ*^2^ values for each type of interatomic coordination, the *σ*^2^ values after a complete discharge–charge cycle at re-dc 3.5 V approached their original values with small deviations, indicating the high reversibility of the lithium intercalation/deintercalation sites and structural flexibility, which is consistent with the results of XANES.^49^ The effects of the lithium-intercalation/deintercalation-driven phase transition on the manganese and nickel species were further identified as well. From the OCV to dc 1.8 V, the *σ*^2^ values of Mn–O and Mn–TM increased, especially that of Mn–TM, indicating that the lithium intercalation increased the systematic disorder of the Mn–O and Mn–TM coordination, leading to the occurrence of the phase transition from LNMO to L_2_NMO. After dc 1.8 V, the charging process to re-ch 4.1 V and re-ch 4.9 V caused a delithiation process that reduced the systematic disorder of the Mn–O and Mn–TM to the original *σ*^2^ values with slight deviations, completing the lithiation–delithiation cycle. In terms of the nickel species, the *σ*^2^ values of Ni–O and Ni–TM at each potential were almost same as those at the OCV, except at re-ch 4.9 V. At this relatively high charging potential, the lithium was extracted from the nickel sites, resulting in increased nickel valence and Ni–O disorder. The variations in the *σ*^2^ values of Mn/Ni–O and Mn/Ni–TM at different potentials are consistent with the results of the XAS spectra edge-positions and *in situ* XRD patterns.

In order to schematically present the structural variations of the first lithium intercalation/deintercalation sites (Mn–O and Mn–TM) in LNMO during a discharge–charge cycle, the wavelet transform (WT) spectra of the Mn K-edge are displayed. Fig. 2g shows two red regions in each spectrum; the one with a shorter radius distance (*R*) was assigned to Mn–O (first shell), and the one with a longer radius distance was assigned to Mn–TM (second shell). In general, a target metal atom preferentially coordinates with a neighboring atom with lower atomic order in the first shell, and then coordinates with one with higher atomic order in the second shell. The magnitudes of Mn–O and Mn–TM at dc 1.8 V were weaker than those at the OCV, which was attributed to the covalent effect in the lithium-intercalation-driven phase transition between LNMO and L_2_NMO. Once LNMO underwent the recharging process, the lithium deintercalated from the crystal lattice, resulting in the magnitudes of Mn–O and Mn–TM returning to the original state at OCV with small deviations. The magnitude variation of Mn–O and Mn–TM during the discharge–charge cycle indicates that the covalent effect only drives the lithium-intercalation-driven phase transition, and that the change in the crystal lattice structure is reversible. Furthermore, the differences among dc 1.8 V, re-ch 4.1 V, and OCV were further confirmed from the colour 3D WT-EXAFS spectra in Fig. 2h. The difference in magnitude between the dc 1.8 V and OCV states is obvious, as indicated by the blue areas, but the difference in magnitude between the re-ch 4.1 V and OCV states is not obvious, even though it has been multiplied 10 times.

**Fig. 3:**
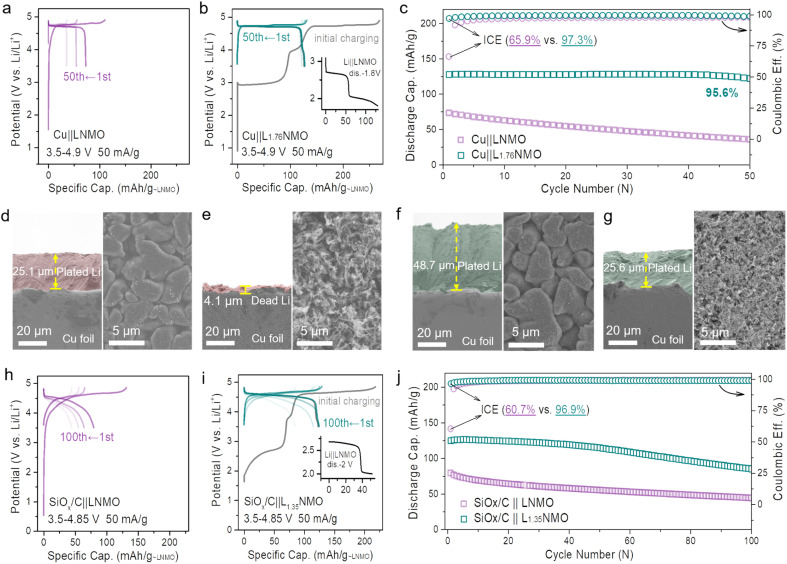
Performance of the anode-free and SiO_*x*_/C coin-type full cell with pre-lithiation in Li_*x*_NMO. Voltage profiles of (a) the Cu‖LNMO anode-free coin cell and (b) the Cu‖L_1.76_NMO anode-free coin cell. (c) Cycling performance of (a) and (b). Cross-sectional SEM images (left) and surface SEM images (right) of Cu foil after (d) 5 mA h cm^−2^ Li plating, (e) 5 mA h cm^−2^ Li plating and then 5 mA h cm^−2^ Li stripping, (f) 10 mA h cm^−2^ Li plating, (g) 10 mA h cm^−2^ Li plating and then 5 mA h cm^−2^ Li stripping at 1 mA cm^−2^. Voltage profiles of (h) SiO_*x*_/C‖LNMO full-cell and (i) SiO_*x*_/C‖L_1.35_NMO full-cell. (j) Cycling performance of (h) and (i). (L_1.76_NMO and L_1.35_NMO cathodes were prepared *via* the electrochemical pre-lithiation method.)

Based on the aforementioned considerations, the cathode pre-lithiation strategy, which can store extra Li^+^ in the LNMO structure, has been demonstrated. To construct an anode-free cell with a long cycling life and high energy density, we combined the cathode pre-lithiation and a complementary fluorine-containing electrolyte strategy to extend the lifespan of the anode-free cell. Over-lithiated L_1.76_NMO was prepared *via* the electrochemical lithiation of LNMO, and then a Cu‖L_1.76_NMO anode-free coin cell was assembled with a fluorine-containing electrolyte (Fig. 3b). Compared with the Cu‖LNMO anode-free coin cell (Fig. 3a), the Cu‖L_1.76_NMO anode-free coin cell exhibits a higher initial specific capacity of 129 mA h g^−1^. The application of the fluorine-containing electrolyte not only improves the ICE (from 65.9% to 97.3%) but also results in a higher CR of 95.6% after 50 cycles (Fig. 3c). The extra active Li^+^ is stored in the over-lithiated L_1.76_NMO, and then all the active Li^+^ transfers from L_1.76_NMO to Cu during the initial charging process, directly supplementing/offsetting the irreversible Li loss in the initial and subsequent cycles.^50,51^ Meanwhile, the fluorine-containing electrolyte enhances the reversibility of Li plating/stripping, thereby extending the lifespan of the anode-free cell.

To investigate the nucleation of Li on the Cu substrate, a Cu cathode and Li anode were constructed as half-cells. Fig. 3d shows the SEM images of Cu after 5 mA h cm^−2^ Li plating, which is akin to LNMO depositing all the Li onto the Cu. Fig. 3e shows the SEM images of Cu after 5 mA h cm^−2^ Li plating and 5 mA h cm^−2^ Li stripping, which is equivalent to the Cu‖LNMO AF-LMB after first charge–discharge process. In such a case, vast voids can be observed on the Cu surface of the Cu‖LNMO AF-LMB, which can easily generate “dead Li” during the Li stripping process and facilitate the generation of Li dendrites.^52^Fig. 3f shows the SEM images of Cu after 10 mA h cm^−2^ Li plating, which is akin to L_2_NMO depositing all the Li onto the Cu. Fig. 3g shows the SEM images of Cu foil after 10 mA h cm^−2^ Li plating and 5 mA h cm^−2^ Li stripping. In this case, the Li plating/striping on Cu is similar to that of the Cu‖L_2_NMO AF-LMB re-discharged to 3.5 V. Part of the Li is stored in the Cu anode to offset the formation of the SEI, and the remaining Li is involved in the subsequent cycles. In contrast, although the Cu surface of the pre-lithiated AF-LMB is also partially fragmented, it is much more uniform, which contributes to the subsequent electroplating behavior (Fig. 3e and g right).^7,53^ The cross-sectional image also shows that there is thick Li deposition on the surface of Cu (Fig. 3g left). In addition, comparing the CR and CE of Cu‖L_1.76_NMO and Cu‖LNMO AF-LMBs (Fig. S13†), the results serve to show that the pre-lithiation effectively improves the ICE and reduces the irreversible Li loss during the first charge–discharge process, resulting in better cycle life, but inevitable capacity fading.^21,54^ Compared with LNMO, L_2_NMO deposited twice as much Li on Cu (Fig. 3d and f), but there was no difference in the surface, indicating that the amount of Li plating did not affect the surface morphology of Cu. Therefore, the poor cycle stability of the AF-LMB is mainly caused by the Cu anode, and the reversibility of Li plating/striping on Cu anode determines the lifespan of the AF-LMB.^9^ To improve the reversibility of Li plating on Cu, we improved the ACE of AF-LMB by optimizing the electrolyte.^1^ However, as can be seen from Fig. S3 and S14,† even if the ACE is improved and the electrolyte is optimized, the capacity decay of the AF-LMB is still shocking when the ICE is low. Hence, to construct an anode-free cell with long cycling life and high energy density, we combined the cathode pre-lithiation and complementary fluorine-containing electrolyte strategy to extend the lifespan of the anode-free cell.

The cathode pre-lithiation strategies of are not only suitable for anode-free cells, but also applicable to the Si-based full-cell. The volume change of the Si-based anode during cycling will lead to significant and irreversible electrode pulverization and reorganization of the SEI.^55,56^ The continuous growth of the SEI will lead to limited active Li^+^ loss, resulting in severe initial irreversible capacity loss and low ICE (Fig. S15†). To date, the anode pre-lithiation strategy has mainly been used to solve the above problems for the Si-based full-cell.^17^ In this work, we adopt the cathode pre-lithiation strategy to improve the electrochemical performance of the Si-based full-cell. In the same way, over-lithiated L_1.35_NMO was prepared *via* the electrochemical lithiation of LNMO, and then a SiO_*x*_/C‖L_1.35_NMO full-cell was assembled (Fig. 3i). Compared with the SiO_*x*_/C‖LNMO full-cell (Fig. 3h), the SiO_*x*_/C‖L_1.35_NMO full-cell exhibits a higher initial specific capacity of 124 mA h g^−1^. The extra active Li^+^ offset the initial irreversible capacity loss, significantly improving the ICE (from 60% to 96.5%) and the electrochemical performance of the Si-based full-cell (Fig. 3j).

The electrochemical pre-lithiation method requires (dis)assembly of the battery, making the production process intricate and resulting in the process being difficult to industrialize. At present, an alternative pre-lithiation cathode strategy using ultra-thin lithium foil has been proposed. Ultra-thin lithium foils are adhered to the cathode by rolling. When the lithium foil is in contact with the cathode *via* the electrolyte, they spontaneously generate the over-lithiated cathode through electrochemical reaction, and the degree of lithiation can be adjusted *via* the thickness of the lithium foil (5–10 μm).^35^ In addition, in practical factory production, stable lithium metal powder is generally mixed into the material for pre-lithiation.^39^ However, it is difficult to control the degree of pre-lithiation by controlling the thickness of the ultra-thin lithium foils, and additionally, ultra-thin lithium foils are expensive. Nevertheless, most of these methods have been applied to Si-based and graphite-based anodes rather than to Cu. Therefore, we introduce a convenient engineering method, contact pre-lithiation, and put it to use in cathode pre-lithiation.

**Fig. 4:**
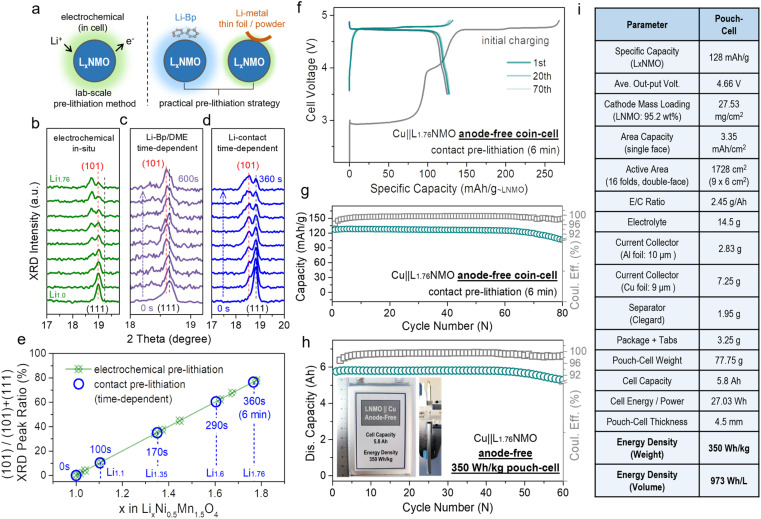
Practical pre-lithiation strategies on L_*x*_NMO and related anode-free cell performance in coin/pouch-cells. (a) Schematic of the electrochemical pre-lithiation, contact pre-lithiation and Li-Bp/DME pre-lithiation. (b) *In situ* XRD patterns of the electrochemical pre-lithiation Li‖LNMO half-cell discharged from OCV to 1.8 V. *In situ* XRD tests of (c) Li-Bp/DME pre-lithiation and (d) contact pre-lithiation, which depend on time, were performed to observe the change of the main peak. By comparing the changes of the (111) main peak in the *in situ* XRD patterns of the three pre-lithiation methods, the feasibility of contact or Li-Bp/DME pre-lithiation and the relationship between the changes in the main peak and time were revealed. (e) Ratio of the (101) peak area corresponding to different *x* values in L_*x*_NMO (0 ≤ *x* < 2) (according to the *in situ* XRD patterns in Fig. 4b and d). Comparison of the curves of the electrochemical pre-lithiation method (green) and contact pre-lithiation method (blue) shows that both methods have an outstanding linear relationship. (f) Voltage profiles of the Cu‖LNMO (6 min) anode-free coin cell. (g) Cycling performance of the Cu‖LNMO (6 min) anode-free coin cell. (h) Capacity and coulombic efficiency at different cycle numbers for the Cu‖LNMO anode-free pouch cell, and a photograph of the 350 W h kg^−1^ (973 W h L^−1^) level anode-free pouch cell. (i) Parameters for the Cu‖LNMO anode-free pouch cell. (An LNMO (6 min) cathode was prepared by the contact lithiation method by regulating the short circuit time.)

Fig. 4a presents a schematic diagram of the contact pre-lithiation method. The lithium foil is in direct contact with the LNMO cathode through the electrolyte. Due to the lowest potential of lithium among the metallic anodes, under the reaction of the potential difference, when the cathode is in contact with lithium, the lithium is converted into lithium ions, and the electrons move to the cathode spontaneously, accompanied by lithium ions.^57^ The lithium ions are intercalated into the cathode material, completing the contact pre-lithiation. To verify the feasibility of the contact pre-lithiation method, *in situ* XRD measurements of the electrochemical pre-lithiation (Fig. 4b) and contact pre-lithiation (Fig. 4d) of LNMO were conducted.

In electrochemical pre-lithiation, the (111) peak of LNMO gradually split into two peaks during the discharge to 1.8 V, and the lower-angle peak is the (101) peak of L_2_NMO, indicating the further lithiation and the generation of L_2_NMO. The LNMO using the contact pre-lithiation method showed the same trend as in the electrochemical lithiation; the (101) peak of L_2_NMO also appeared with increasing contact time, indicating the feasibility of contact pre-lithiation. By controlling the contact time, the value of *x* in L_*x*_NMO (0 ≤ *x* < 2) can be precisely regulated (Fig. S16†). When the contact time reached 6 min, comparison of the calculated ratios of the peak areas of (101) and (111) showed that the value was obtained using the contact pre-lithiation method was the same as that obtained with the electrochemical pre-lithiation (Fig. 4e). Li-biphenyl (Li-Bp) has a highly reducing property with a redox potential of 0.33 V; therefore, Li-Bp has the potential to be a stronger pre-lithiation reducing agent. When the LNMO cathode is in contact with the Li-Bp complex, the LNMO will be reduced to L_*x*_NMO (1 < *x* < 2), and the lithium ions will intercalate into the LNMO cathode to achieve charge compensation, accompanied by the reduction of Mn^4+^ to Mn^3+^. *In situ* XRD measurements of Li-biphenyl pre-lithiation (the preparation method for the pre-lithiation agent is shown in Fig. S17†) were also conducted (Fig. 4c), and the peak changes showed the same trend as in electrochemical lithiation, indicating the feasibility of Li-biphenyl pre-lithiation (denoted as Li-Bp/DME pre-lithiation).^28^ The rigid contact pre-lithiation method will put the whole electrode material (especially thick electrode materials) at risk of over-lithiation and concentration gradients. However, the excellent transfer coefficients of spinel materials and gentler liquid-phase Li-Bp pre-lithiation can alleviate the over-lithiation and the structural damage caused by the chemical impact of the lithiation agent to a certain extent. Fig. 4f shows the charge–discharge curve of Cu‖L_1.76_NMO AF-LMB after contact pre-lithiation for 6 min; the discharge capacity of the electrode prepared using the contact pre-lithiation method is almost the same as that of the electrode prepared by the electrochemical pre-lithiation. In addition, the Cu‖L_1.76_NMO AF-LMB exhibits exceptional cycling stability with a high CR of 94% after 70 cycles (Fig. 4g). The *in situ* XRD and electrochemical performance test showed that the contact pre-lithiation method also did not affect the structure of LNMO. However, after 70 cycles, the capacity decays sharply due to the Li plated on the Cu in the first discharge gradually transforming into “dead Li”, thus causing the subsequent capacity decay (Fig. S18†).

Meanwhile, an anode-free pouch cell was assembled using L_1.76_NMO (prepared by the contact pre-lithiation method) with Cu (Fig. 4h). The detailed parameters of the pouch cell are shown in Fig. 4i. Moreover, this 350 W h kg^−1^ (973 W h L^−1^) pouch cell showed no drop in output capacity with a high CR of 97% after 50 cycles. However, a significant ‘knee point’ can be observed at around 50 cycles, at which the well-maintained CR (before 50 cycles) starts to decline. The subsequent capacity decay of the anode-free pouch cell is ascribed to the Cu anode (Fig. 1b and S4†). To be honest, due to our limited skill in the fabrication of pouch-type cells, there remains a lot of scope to improve the energy density and cycle stability at the cell level.

## Conclusions

To conclude, a high-energy and long-lifespan anode-free lithium metal battery was successfully achieved using a pre-lithiated Li-rich Li_2_Ni_0.5_Mn_1.5_O_4_ cathode. The reversible phase transition between typical spinel LiNi_0.5_Mn_1.5_O_4_ and Li-rich spinel Li_2_Ni_0.5_Mn_1.5_O_4_ is demonstrated by a series of systematic characterizations (*e.g.*, *in situ* XRD, HR-TEM and XAS), which indicated that the crystallographic vacancies of LiNi_0.5_Mn_1.5_O_4_ can be occupied by extra Li^+^ to form over-lithiated Li_2_Ni_0.5_Mn_1.5_O_4_. After precisely regulating the Li-rich degree using the electrochemical method, the over-lithiated Li-rich Li_1.76_Ni_0.5_Mn_1.5_O_4_ cathode was harvested, which can provide an additional 0.76 mol of excess Li^+^ in the initial charging process to offset the irreversible capacity loss, resulting in enhancement of the cycling retention without energy density decay. Moreover, the introduction of a fluorine-containing electrolyte can further improve the average coulombic efficiency and the cycling performance of the anode-free cell. Notably, more practical pre-lithiation strategies have been demonstrated and realized in terms of engineering, *e.g.*, Li-metal contact pre-lithiation and Li-biphenyl immersing pre-lithiation methods. By applying the contact pre-lithiation method, an anode-free pouch cell consisting of Li_2_Ni_0.5_Mn_1.5_O_4_ cathode and Cu anode was fabricated and delivered a high energy density of 350 W h kg^−1^ (973 W h L^−1^), as well as a long lifespan, retaining 97% capacity after 50 cycles. This design realizes the pre-lithiation of a high-voltage spinel cathode at the engineering level and provides a prospect for the practical application of the anode-free cell with high gravimetric and volumetric energy density.

## Data availability

All the data supporting this study are included in the manuscript and the ESI.† Example data sets are available on request.

## Author contributions

L. C., X. K. and Y. Q. contributed to the design of the research and performed the experimental data analysis. L. C. conducted the materials synthesis, electrochemistry and cell performance. C. C. and Y. L. conducted the XAS experiments and the analysis of XAS results. X. W. and T. L. conducted the SEM experiments and related data analysis. Y. T., G. Z. and B. Z. helped to conduct the XRD experiment and FAULTS simulation. S. Z. conducted the HR-TEM experiment. H. Z. and Y. Y. drew the schematics. X. K., Y. L. and Y. Q. supervised the work. All authors discussed the results, co-wrote and commented on the manuscript.

## Conflicts of interest

The authors declare no competing financial interests.

## Supplementary Material

SC-014-D2SC06772B-s001
