# Supplementary material for: Prolonged lifespan of initial-anode-free lithium-metal battery by pre-lithiation in Li-rich Li2Ni0.5Mn1.5O4 spinel cathode
Source: Chem Sci. 2023 Jan 25;14(8):2183–91. doi: 10.1039/d2sc06772b (PMC9944687; doi:10.1039/d2sc06772b)
Supplement: SC-014-D2SC06772B-s001 [file SC-014-D2SC06772B-s001.pdf]

Electronic Supplementary Material (ESI) for Chemical Science.
This journal is © The Royal Society of Chemistry 2023

Supporting Information of

Prolong lifespan of initial-anode-free lithium-metal battery by pre-lithiation in Li-rich $Li_2Ni_{0.5}Mn_{1.5}O_4$ spinel cathode

Leiyu Chen,[a]‡ Chao-Lung Chiang,[b]‡ Xiaohong Wu,[a] Yonglin Tang,[a] Guifan Zeng,[a] Shiyuan Zhou,[a] Baodan Zhang,[a] Haitang Zhang,[a] Yawen Yan,[a] Tingting Liu,[d] Hong-Gang Liao,[a] Xiaoxiao Kuai,*[a,c] Yan-Gu Lin,*[b] Yu Qiao,*[a, c] Shi-Gang Sun[a]

[a] State Key Laboratory of Physical Chemistry of Solid Surfaces, *i*ChEM (Collaborative Innovation Center of Chemistry for Energy Materials), Department of Chemistry, College of Chemistry and Chemical Engineering

Xiamen University

Xiamen 361005, PR China

E-mail: kuaixiaoxiao@xmu.edu.cn; yuqiao@xmu.edu.cn

[b] National Synchrotron Radiation Research Center

Hsinchu 30076 Taiwan, R.O.C

E-mail: lin.yg@nsrrc.org.tw

[c] Fujian Science & Technology Innovation Laboratory for Energy Materials of China (Tan Kah Kee Innovation Laboratory)

Xiamen University

Xiamen 361005, PR China

E-mail: kuaixiaoxiao@xmu.edu.cn; yuqiao@xmu.edu.cn

[d] School of Environmental Science and Engineering
Suzhou University of Science and Technology
Suzhou 215009, China

* Corresponding authors: kuaixiaoxiao@xmu.edu.cn (X. K.); lin.yg@nsrrc.org.tw (Y. L.); yuqiao@xmu.edu.cn (Y. Q.)

‡ L. Chen and C.-L. Chiang contributed equally to this work.

Experimental Section

Preparation of $LiNi_{0.5}Mn_{1.5}O_4$(LNMO) cathode

LNMO (Guangdong Canrd New Energy Technology Co., Ltd.) was heated at 700 °C for 20 h in air to obtain the *Fd-3m* type LNMO. The heating rate was 5 °C/min while cooling was uncontrolled. To fabricate LNMO cathode, LNMO with *Fd-3m* structure, carbon black powder (Super P, Timcal), polyvinylidene difluoride (PVDF, Alfa Aesar Co., Ltd.) with a weight ratio of 80:10:10 in N-methyl pyrrolidone (NMP, Guangdong Canrd New Energy Technology Co., Ltd.) were mixed in SK-300SII CE mixing machine for 25 min, producing a black slurry. Then, black slurry was spread on a clean carbon coated aluminum foil (Suzhou Sinero Technology Co., Ltd.) by a doctor blade. After solvent drying, the loaded foil was roll-pressed and was punched into disk followed by additional vacuum drying at 110 °C for 12 h. The mass of LNMO cathode is 6 mg/cm^2 (Li||LNMO half-cell) or 7 mg/cm^2 (SiO_x/C||LNMO full-cell) and 9 mg/cm^2 (Cu||LNMO anode-free cell) or 27.53 mg/cm^2 (Cu||LNMO anode-free pouch cell) on average, respectively. Then, the LNMO cathodes were punched into discs with a diameter of 11 mm, while the 27.53 mg/cm^2 LNMO cathode was cut into 30*40 mm^2 plates.

Preparation of $Li_xNi_{0.5}Mn_{1.5}O_4$(L_xNMO) cathode

L_xNMO ($0 \leq x < 2$) cathodes were fabricated by electrochemical pre-lithiation, contact pre-lithiation and Li-Biphenyl (Li-Bp) pre-lithiation method. As for electrochemical pre-lithiation method, L_xNMO cathodes were prepared by over discharge of LNMO cathodes in Li||LNMO half-cells, since there are sufficient Li resources on the counter electrode. The x vale can be

controlled by the discharging cut-off voltage. As for contact pre-lithiation method, the lithium metal sheet (China Energy Lithium Co., Ltd.) contacted directly with the LNMO cathode which saturated with as-prepared electrolytes, then L_xNMO cathodes were obtained with different x vale which can be controlled by contact time. As for Li-Bp/DME pre-lithiation method, 0.005mol of biphenyl (Bp) was dissolved in 10ml of 1,2-Dimethoxyethane (DME), then 0.005mol of chopped lithium foil were added, and 0.5mol/L Li-Bp/DME solution was obtained after 12h of reaction at room temperature, all operations were performed in an argon-filled glove box (H_2O <0.01 ppm, O_2 <0.01 ppm). L_xNMO cathode was obtained by the redox reaction between LNMO cathode and Li-Bp-DME solution, in which LNMO cathode was immersed in 400 μL 0.5mol/L Li-Bp-DME solution, and the reaction time was controlled different minutes. L_xNMO cathodes with different lithium contents can be obtained ($0 \leq x < 2$).

Preparation of SiO_x/C electrode

To fabricate SiO_x/C electrode, SiO_x/C (Shanghai Aladdin Bio-Chem Technology Co., Ltd.), carbon black powder, carboxymethyl cellulose (CMC, Shanghai Aladdin Bio-Chem Technology Co., Ltd.), polyacrylic acid (PAA, Shanghai Aladdin Bio-Chem Technology Co., Ltd.) with a weight ratio of 70:15:7.5:7.5 in deionized water were mixed in SK-300SII CE mixing machine for 30 min, producing a black slurry. Then, black slurry was spread on Cu foil (MTI Co., Ltd.) by a doctor blade. After solvent drying, the loaded foil was roll-pressed and was punched into disk followed by additional vacuum drying at 80 °C for 12 h. The mass of SiO_x/C electrodes is 8

mg/cm^2 (Li||SiO_x/C half-cell) or 12 mg/cm^2 (SiO_x/C||LNMO full-cell) on average, respectively. Then, the SiO_x/C electrodes were punched into discs with a diameter of 11 mm or 13 mm, respectively.

Preparation of half-cells, full-cells and in-situ cell

Li||LNMO half-cells were assembled in CR2032-type coin cells with LNMO cathodes as working electrodes, lithium metal sheet as counter electrodes, Celgard 2325 (Whatman Co., Ltd.) as separator, and 1 M $LiPF_6$ in ethyl carbonate/ethyl methyl carbonate (EC/EMC) with a ratio of 3:7 in volume as the electrolyte (base electrolyte, Suzhou Duoduo Chemical Technology Co., Ltd). Li||SiO_x/C half-cells and SiO_x/C||LNMO full-cells were assembled using a similar protocol, but using SiO_x/C (11 mm) as working electrode and SiO_x/C (13 mm) as counter electrode, respectively. The Li||LNMO *in-situ* cell was assembled using a similar protocol, but using *in-situ* electrochemical cell.

Preparation of anode-free cells and pouch cell

Cu||LNMO anode-free cells were assembled in CR2032-type coin cells with LNMO cathodes as working electrodes, Cu foil as counter electrodes, Celgard 2325 as separator, and base electrolyte or 1M $LiPF_6$ and 0.02M LiDFOB in fluoroethylene carbonate/hydrogen fluoride-ether/fluoroethylene methyl carbonate (FEC/HFE/FEMC) with a ratio of 2:2:6 in mass as the electrolyte (Fluorine-electrolyte, Suzhou Duoduo Chemical Technology Co., Ltd.). Cu||LNMO anode-free pouch cell was assembled using LNMO (30*40 mm^2) as the cathode, Cu foil (31*41 mm^2), Celgard 2325 as separator, Fluorine-electrolyte as the electrolyte, and Al-plastic film as

packaging.

Characterizations

The X-ray diffraction (XRD) patterns were performed on Rigaku SmartLab-SE Powder X-ray diffractometer fitted with Cu-Kα X-rays (λ =1.5406Å) radiation at a scan rate of 2 °/min in the scan range (2θ) of 10-80 °. The data of *in-situ* XRD for every single pattern was collected for 10 min with scan rage (2θ) of 15-60 °. The high Resolution Transmission electron microscopy (HRTEM) images of LNMO were obtained by JEOL JEM-2100 Plus transmission electron microscope, operated at 200 kV. The morphologies of Li plating/stripping on Cu foil were obtained by Scanning electron microscopy (SEM, ZEISS GeminiSEM 500). The Mn and Ni *K-edge* X-ray absorption spectroscopy (XAS) spectra were collected in transmission mode at beamline 1W1B of the Taiwan Photon Source Beamline (TPS 44A) station of the National Synchrotron Radiation Research Center (NSRRC) in Hsinchu, Taiwan. The different voltage states of LNMO samples and standards were analyzed by using X-ray absorption near-edge structure spectra (XANES). After XANES data processed in Athena software, XANES spectra were normalized for further comparisons. The R-space of samples were Fourier-transformed (FT) from these calibrated and normalized extended X-ray absorption fine structure (EXAFS) spectra to further explore the internal structure. To supplement the EXAFS fitting results, wavelet transform (WT) analysis of Mn *K-edge* spectra is further performed to explore more intuitive changes of the surrounding coordination environments of transition metal (TM).

Electrochemical measurements

Galvanostatic cycling tests were conducted within a voltage window of 3.5-4.9 V (Li||LNMO half-cells/Cu||LNMO anode-free coin cells/Cu||LNMO anode-free pouch-cell) or 3.5-4.85 V (SiO_x/C||LNMO full-cells) using Neware battery cycler (CT-4008T-5 V10 mA-164) at 26 °C. All cells were (dis)charged at 0.5 C (1 C =100 mA/g). For Li||Cu half-cell measurements, 5 or 10 mAh/cm^2 was plated onto bare Cu foil at a current density of 1 mA/cm^2, and then stripped with a fixed capacity of 5 mAh/cm^2 with a cut-off voltage of 1.5 V.

Supplementary Figures/Table

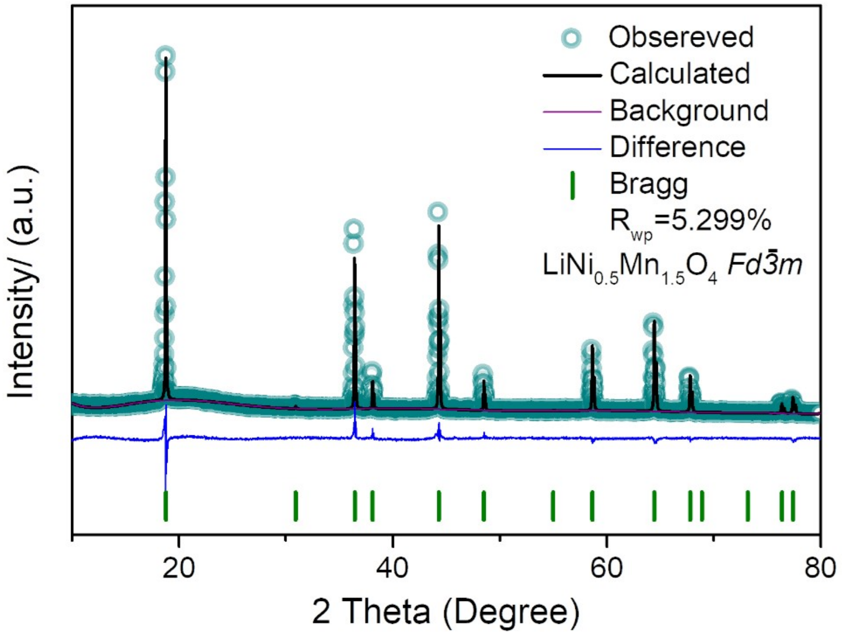

Fig. S1 Representative refinement pattern of *Fd-3m* type LNMO.

Table S1 Rietveld refinement results on the XRD pattern of Fd-3m type LNMO.

Space group Fd-3m.			R $_{wp}$=5.299%	Chi2=2.65	
a (Å)	8.1685(9)			α (°)	90
b (Å)	8.1685(9)			β (°)	90
c (Å)	8.1685(9)			γ (°)	90
Atom	Site	x	y	z	Occ
Li	8a	0.12500	0.12500	0.12500	1
Ni	16d	0.50000	0.50000	0.50000	0.25
Mn	16d	0.50000	0.50000	0.50000	0.75
O	32a	0.26320	0. 26320	0. 26320	1

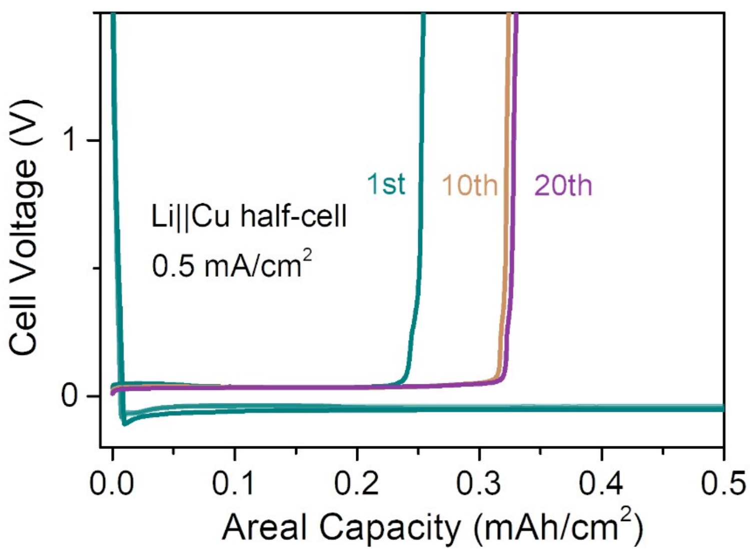

Fig. S2 Voltage profiles of Li||Cu half-cell at 0.5 mA/cm^2 for different cycles.

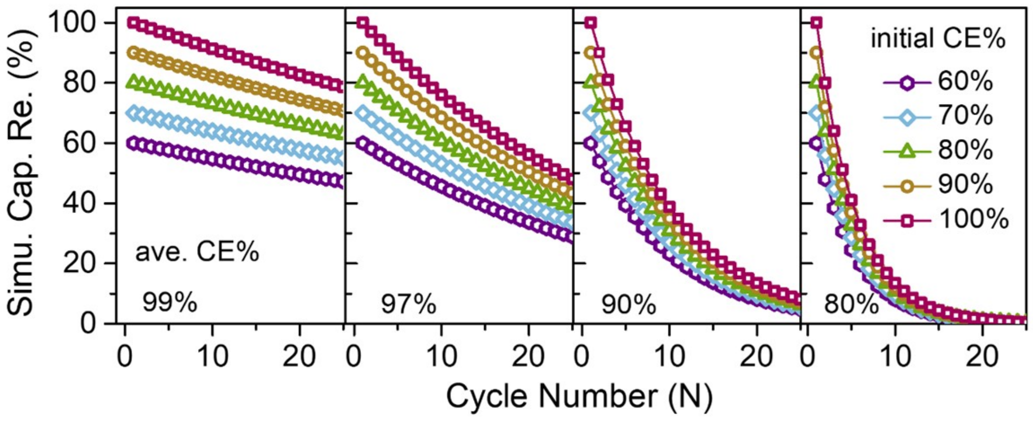

Fig. S3 Simulation of the capacity retention with different initial/average Coulombic efficiency in the anode free cell system.

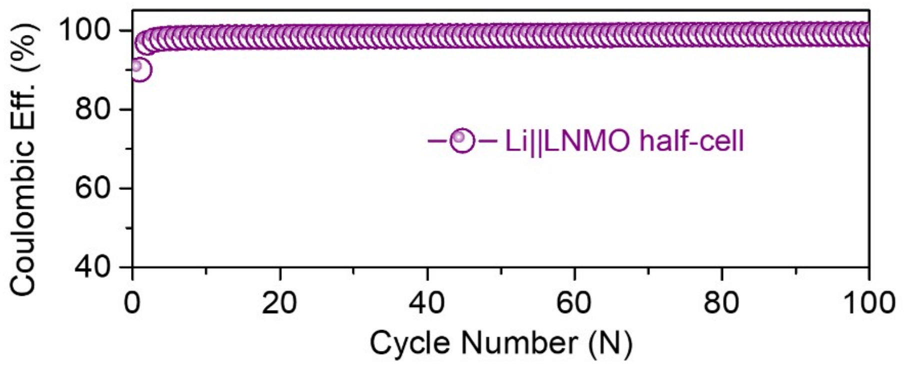

Fig. S4 Cycling performance of Li||LNMO half-cell under a high-voltage window of 3.5-4.9 V at 50 mA/g.

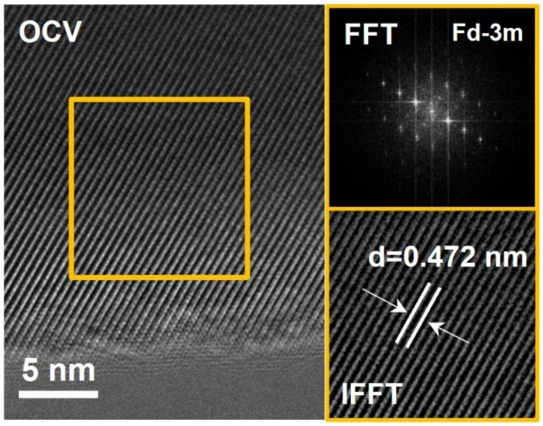

Fig. S5 HRTEM images of OCV state LNMO. Zoomed-in images from selected regions are shown in insets with its corresponding FFT and IFFT images.

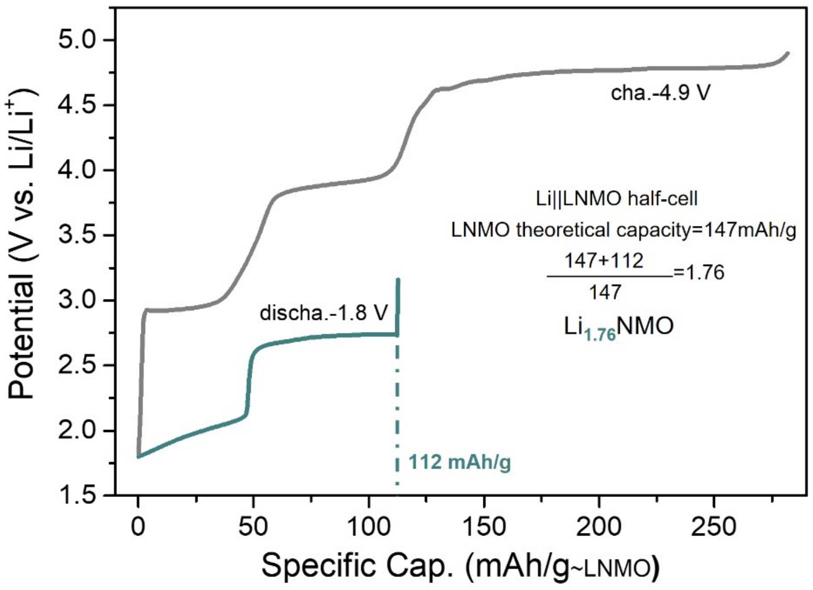

Fig. S6 The charge-discharge curves of Li||LNMO half-cell. The first discharge cut-off potential goes below 1.8 V, followed by subsequent charging to 4.9 V. When controlled the discharging cut-off voltage of Li||LNMO half-cell to 1.8 V, by calculating the amounts of Li^+ intercalating the LNMO structure (The detailed calculation process is shown in Fig. S6), it could obtain $Li_{1.76}Ni_{0.5}Mn_{1.5}O_4$. The detailed structural parameters of $Li_{1.76}Ni_{0.5}Mn_{1.5}O_4$ is shown in Table S2.

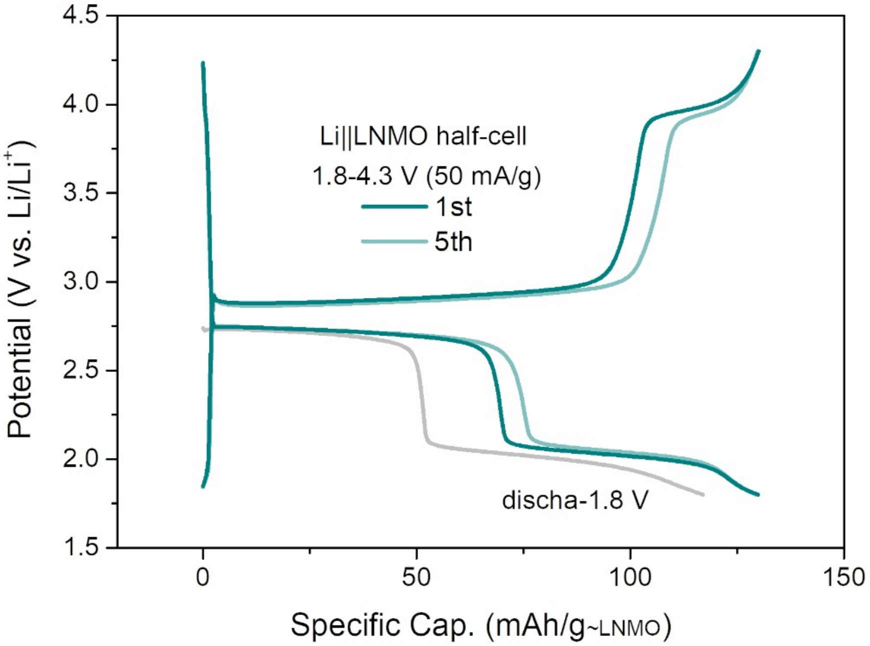

Fig. S7 Voltage profiles of Li||LNMO half-cell between 1.8 V and 4.3 V at 50 mA/g. The Coulombic efficiency of the Li||LNMO half-cell, which first discharged to 1.8 V and then cycled in a voltage range from 1.8 V to 4.3 V, is close to 100%, indicating that the discharge capacity did not come from the reduction of electrolytes. The x value calculated from the discharge capacity is precisely the amount of additional lithium sources from the anode intercalated into the LNMO structure

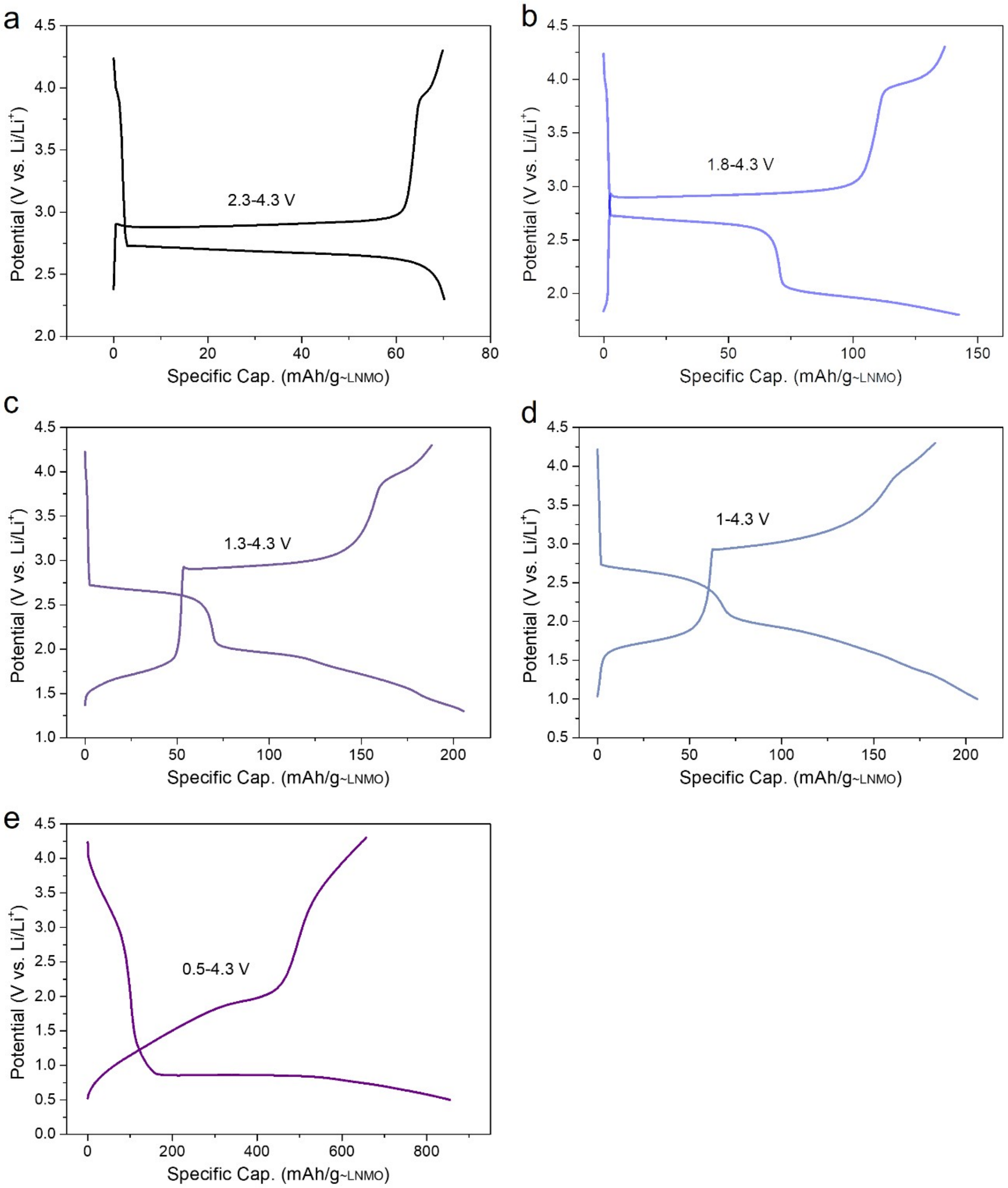

Fig. S8 Voltage profiles of Li||LNMO half-cell under different voltage windows of (a) 2.3-4.3 V, (b) 1.8-4.3 V, (c) 1.3-4.3 V, (d) 1.0-4.3 V, (e) 0.5-4.3 V. According to different voltage lower limits, the Coulomb efficiency is close to 100% when the discharge cut-off potential goes below 2.3 V or 1.8 V, indicating that the LNMO structure is highly reversible. When the discharge cut-off potential drops further, the irreversible capacity will appear, indicating that too much extra

Li^+ intercalating into the crystal leads to the collapse of the LNMO structure, which is unfavorable for subsequent cycles of the anode-free cell. In order to intercalate lithium ions as much as possible, while guaranteeing that the LNMO structure would not be damaged so that the structure could be completely reversible, we choose 1.8 V as the discharge cut-off potential (it could obtain $Li_{1.76}Ni_{0.5}Mn_{1.5}O_4$.). It is essential to extend the lifespan of anode-free cell. It is reasonable and correct that we choose 1.8 V as the discharge cut-off potential.

Table S2 Structural parameters of $Li_{1.76}Ni_{0.5}Mn_{1.5}O_4$.

Composition	Phases	Space group	Percentage
$Li_{\mathbf{1.76}}Ni_{0.5}Mn_{1.5}O_4$	$LiNi_{0.5}Mn_{1.5}O_4$	*Fd-3m*	23.7%
	$Li_{\mathbf{2}}Ni_{0.5}Mn_{1.5}O_4$	$I4_1/amd$	76.3%

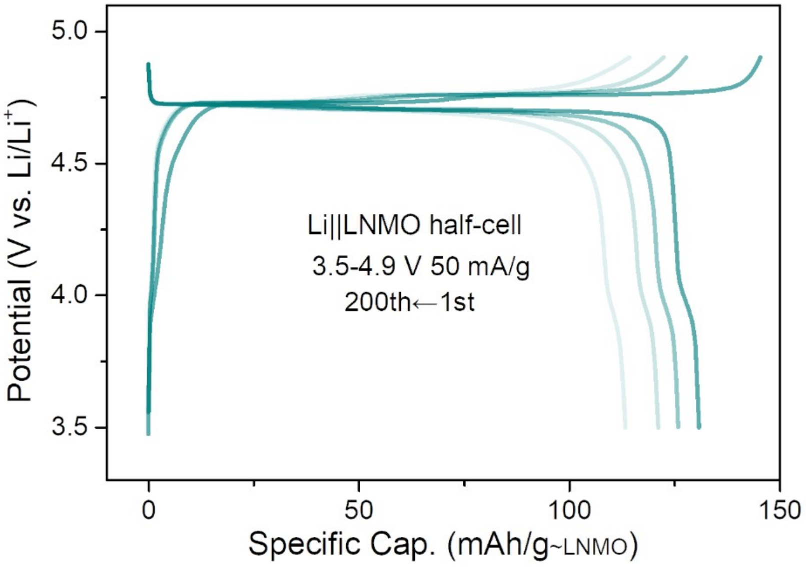

Fig. S9 Voltage profiles of Li||LNMO half-cell between 3.5 V and 4.9 V for long cycles at 50 mA/g (denoted as cell-A).

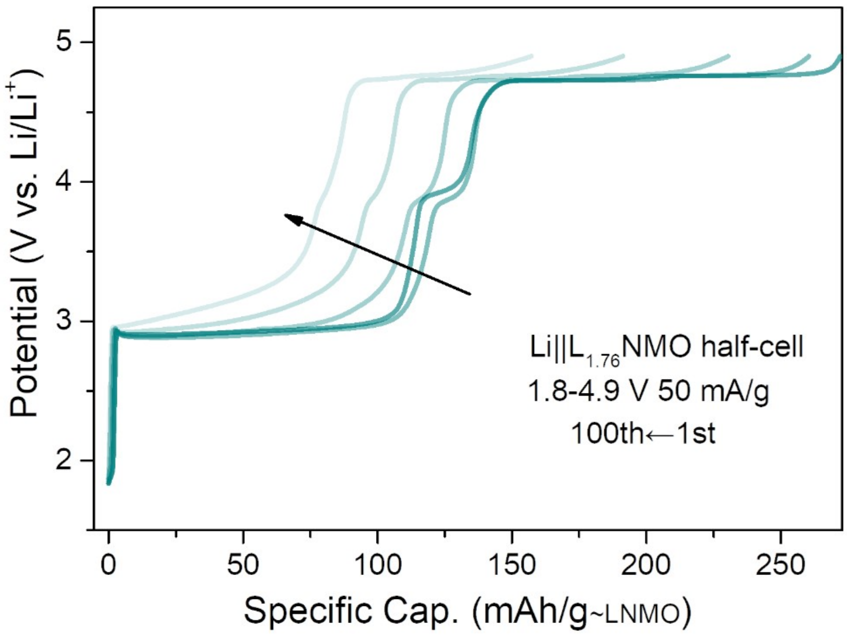

Fig. S10 Voltage profiles of Li||$L_{1.76}$NMO half-cell between 1.8 V and 4.9 V for long cycles at 50 mA/g.

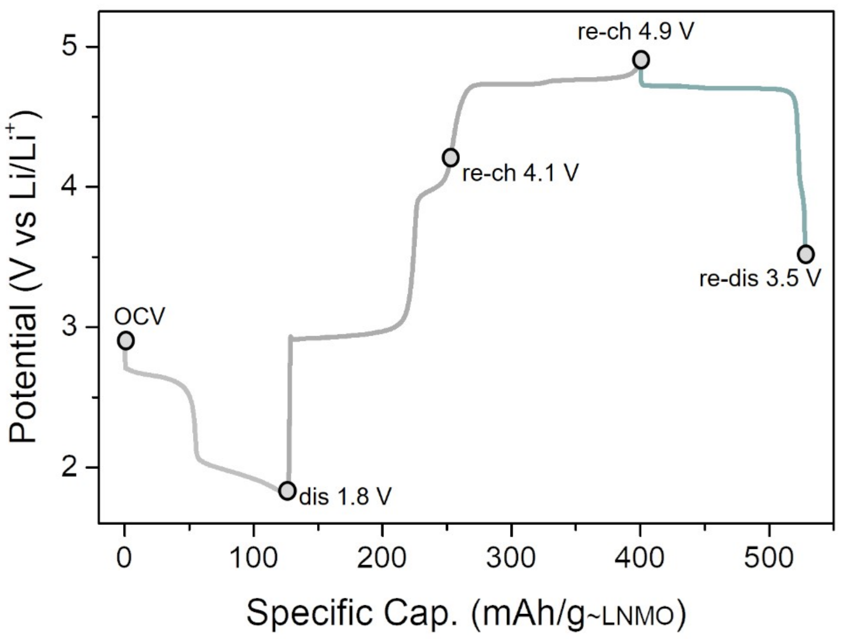

Fig. S11 The corresponding points of the XAS spectra in Mn *K-edge* and Ni *K-edge* for open circuit voltage (OCV), 1.8 V discharged, 4.1 V re-charged, 4.9 V re-charged, and 3.5 V re-discharged states are corresponding to the charge/discharge curves.

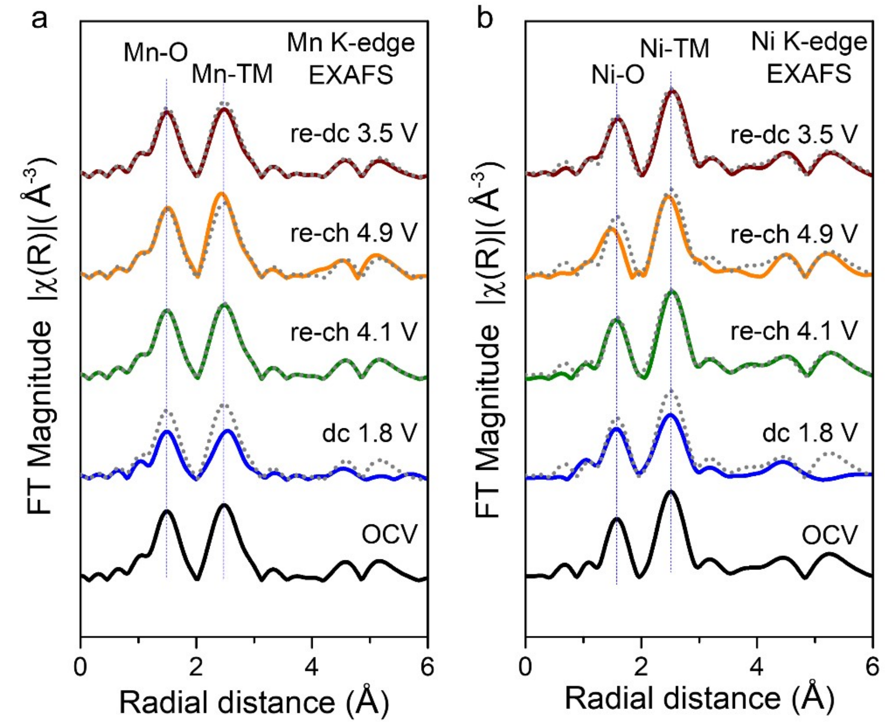

Fig. S12 (a) Mn *K*-edge EXAFS spectra, (b) Ni *K*-edge EXAFS spectra at different voltage states.

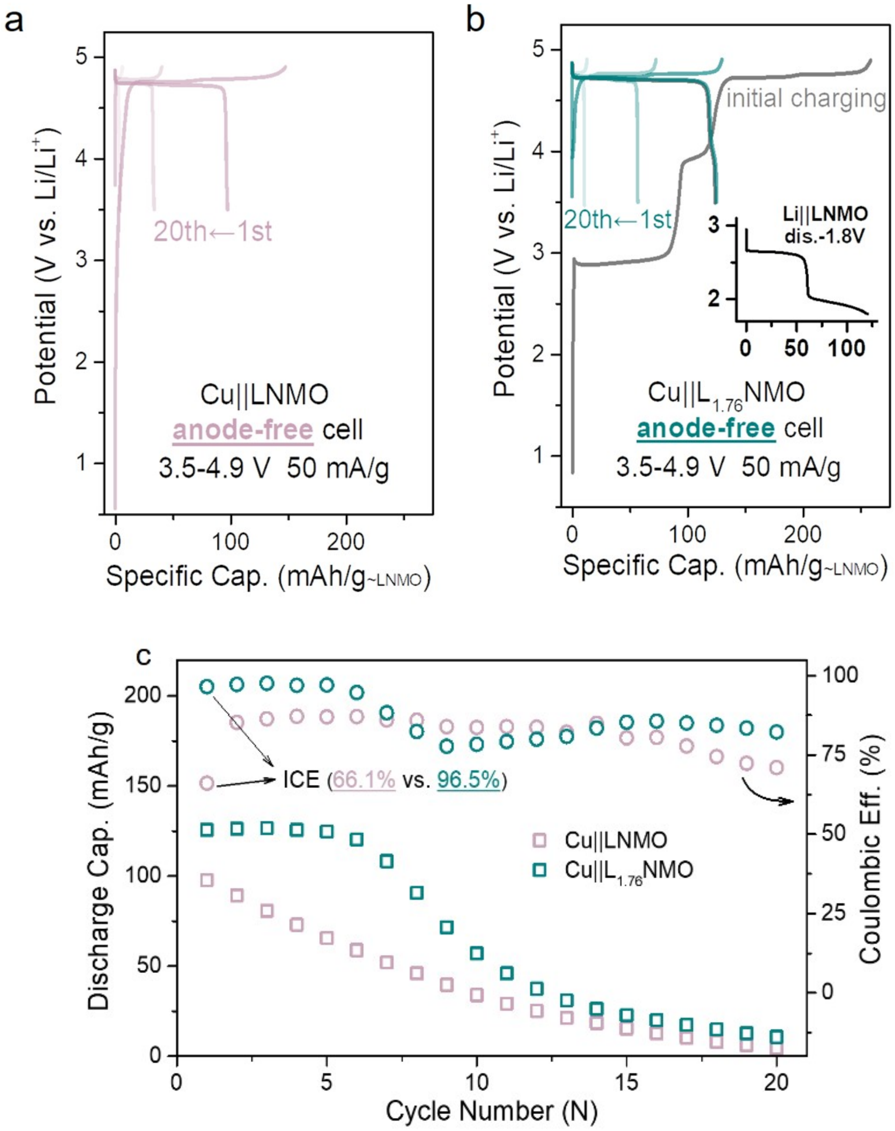

Fig. S13 Voltage profiles of (a) Cu||LNMO anode-free coin cell without pre-lithiation and (b) Cu||$L_{1.76}$NMO anode-free cion cell after pre-lithiation with base electrolyte. (c) Specific cycling performance of (a) and (b).

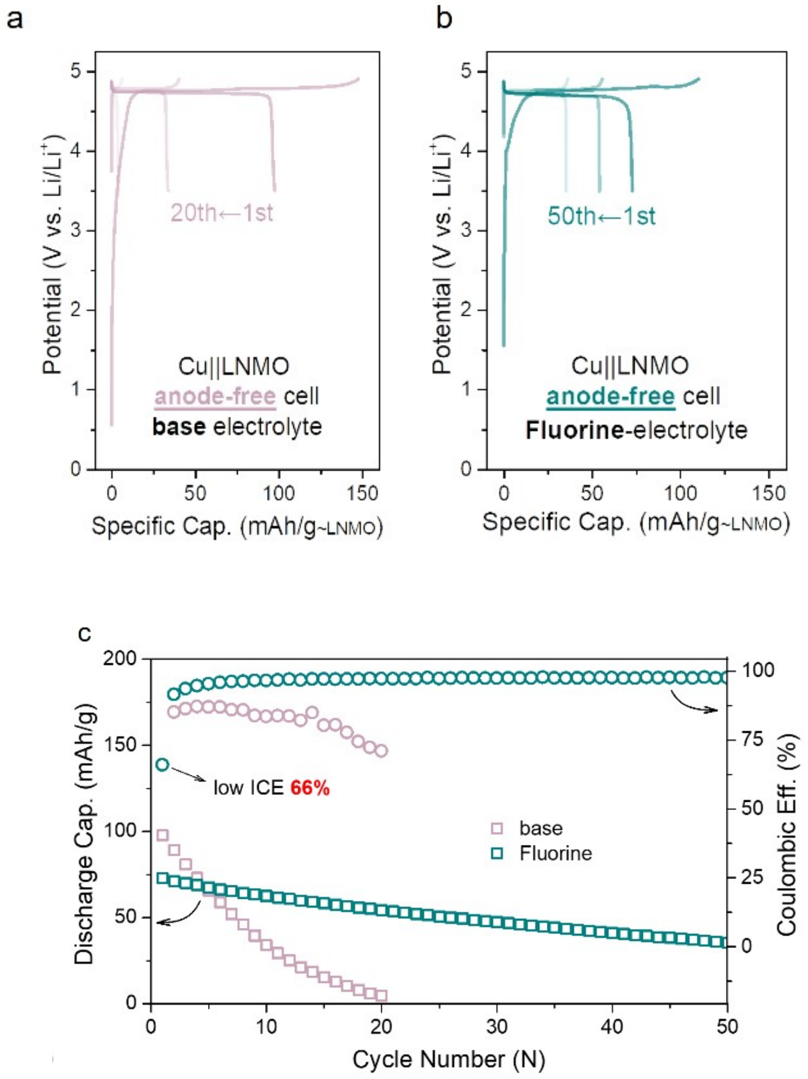

Fig. S14 Voltage profiles of (a) Cu||LNMO anode-free coin cell with base electrolyte and (b) Cu||LNMO anode-free coin cell with Fluorine-electrolyte. (c) Specific cycling performance of (a) and (b).

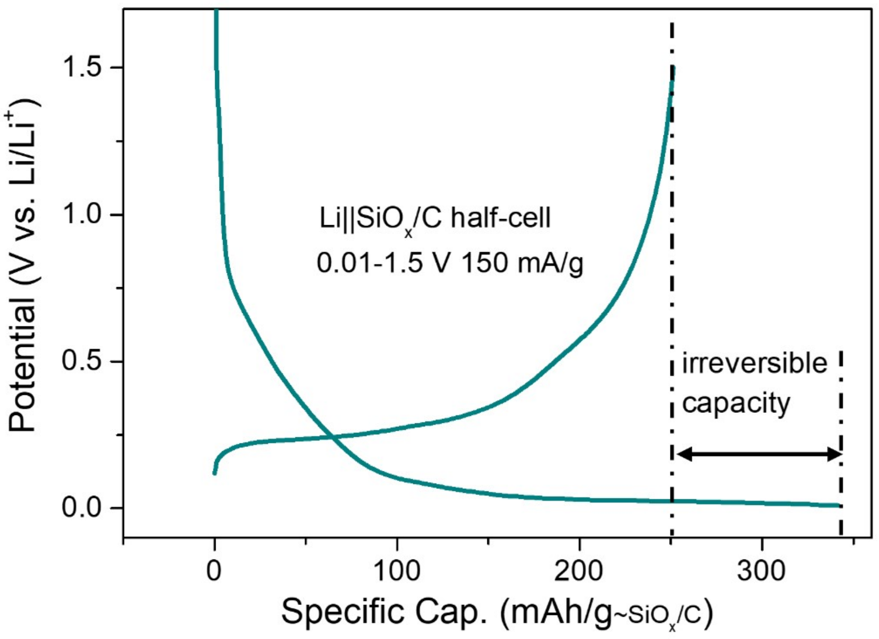

Fig. S15 Voltage profiles of Li||SiO_x/C half-cell for the first cycle. The irreversible capacity can be calculated from charge/discharge specific capacity in the first cycle. By controlling the discharging cut-off voltage of Li||LNMO half-cell, the over-lithiated $L_{1.35}$NMO whose extra Li^+ can offset the irreversible capacity of SiO_x/C was obtained.

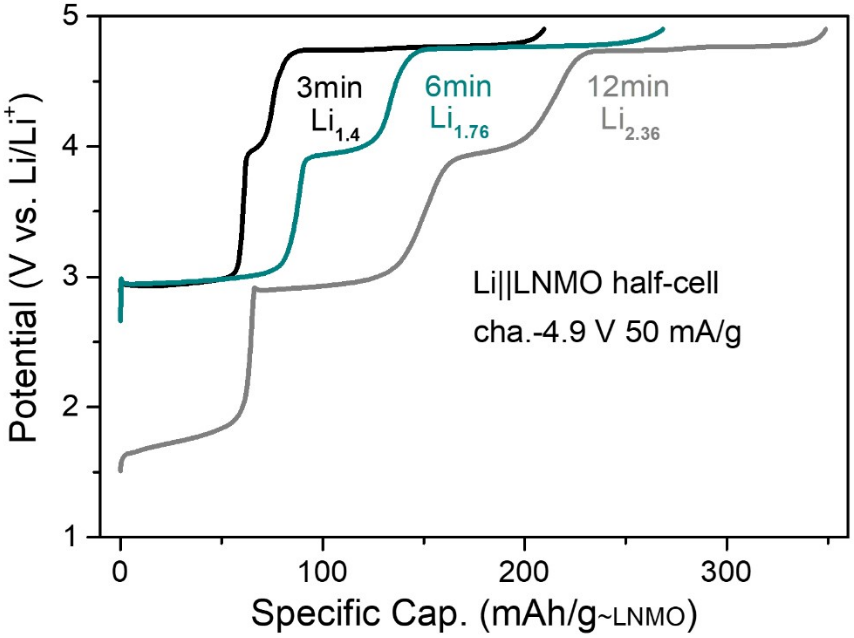

Fig. S16 Voltage profiles of Li||L_xNMO half-cells. L_xNMO cathodes were prepared by contact pre-lithiation method with different contact time. By controlling the contact time, it could regulate precisely different amounts of Li^+ intercalating the LNMO structure.

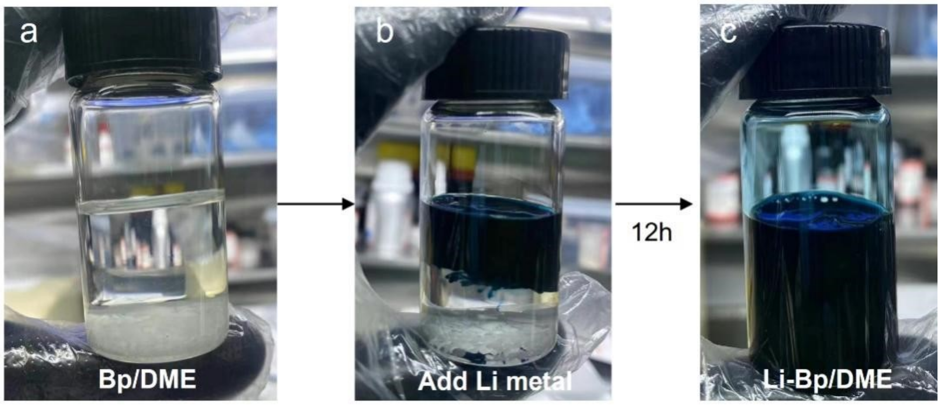

Fig. S17 (a) The solution of biphenyl in 1,2-dimethoxyethane (DME) (denoted as Bp/DME). (b) Dissolving 0.5 molar ratio of Li metal into Bp/DME (0.5M in DME). (c) The pre-lithiation agent Li-Biphenyl/DME (denoted as Li-Bp/DME) turns dark blue after 12h. This indicates the uptake of electron from Li atom to the biphenyl molecular and the formation of biphenyl Li complex (denoted as Li-Bp).

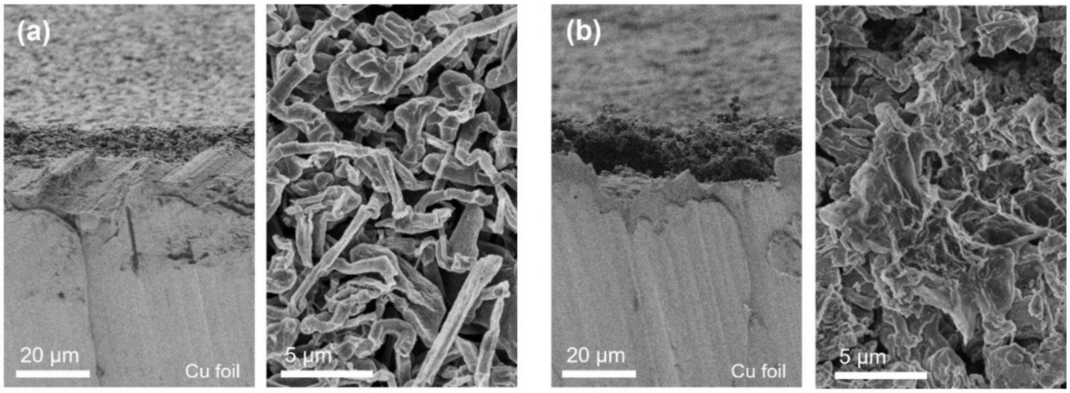

Fig. S18 Cross-section SEM images (left) and surface SEM images (right) of Cu foil after (a) 1 cycle and (b) 70 cycles in Cu||$L_{1.76}$NMO anode-free cell between 3.5 V and 4.9 V at 50 mA/g.
